# A research intelligence approach to assess the research impact of the Dutch university medical centres

**DOI:** 10.1186/s12961-022-00926-y

**Published:** 2022-10-31

**Authors:** Rik Iping, Marielle Kroon, Chantal Steegers, Thed van Leeuwen

**Affiliations:** 1 Research Intelligence and Strategy Unit, Erasmus MC, University Medical Center Rotterdam, Rotterdam, The Netherlands; 2 Leiden University Medical Center (LUMC), Leiden, The Netherlands; 3 Health-RI, Utrecht, The Netherlands; 4 Amsterdam UMC, Amsterdam, The Netherlands; 5 CWTS, Leiden University, Leiden, The Netherlands

**Keywords:** Research intelligence, Bibliometrics, Research impact assessment, Health research policy

## Abstract

**Background:**

The way in which research impact is evaluated and assessed has long been under debate. In recent years the focus is moving away from the use of numerical indicators, towards an emphasis on narratives. The Dutch university medical centres (UMCs) have a long-standing tradition of using bibliometric indicators. Because of the declining interest in indicators alone, this study was designed to repurpose bibliometrics to answer specific strategic questions. In this article we discuss the strategic and policy-based questions, the methodology we used in uncovering relevant information and conclusions we draw from the analyses we performed. The aim of this article is to inform a broader audience about the potential applications of bibliometric information to support a new form of research intelligence.

**Methods:**

In this study we used a curated set of publications from the UMCs. We performed different bibliometric analyses and used bibliometric visualization tools to shed light on research focus, open science practices, collaboration, societal impact and scientific impact.

**Results:**

The analyses allowed us to visualize and contextualize the research focus of the UMCs as a whole, but also to show specific focus areas of each UMC. The UMCs are active in the full spectrum of biomedical research, and at the same time are very complementary to each other. Furthermore, we were able to show the development of open access of UMC publications over time, to support the national mission. Visualizing collaboration is a powerful way of showing both the international orientation and the regional and national engine function of UMCs in research. We were able to assess societal impact by looking at the different channels in which publications find their way to societally relevant sources such as news media, policy documents and guidelines. Finally, we assessed scientific impact and put this into an international perspective.

**Conclusions:**

Research intelligence is able to transform bibliometric information by interpretation and annotation into highly relevant insights that can be used for several different strategic purposes and for research impact assessment in general.

## Background

Research impact assessment is a heavily debated topic. Studies are increasingly addressing research impact assessment, joined by a growing number of conceptual frameworks [1, 2]. These frameworks range from narrative reviews to bibliometric approaches and mixed models, including many aspects that can differ across disciplines based on their relevance. The use of bibliometric indicators has been anchored in research impact assessment for decades. Bibliometrics has focused primarily on quantitative approaches and supplying metrics on citation impact for benchmarking purposes. The Dutch university medical centres (UMCs) have used bibliometrics-based benchmarking reports performed by the Leiden University-based Centre for Science and Technology Studies (CWTS) since 2006. Over the past decade, many developments have placed the application of bibliometric indicators in a different perspective, focusing on responsible use and appropriate valuation (i.e. the Leiden Manifesto^1^, the metric tide,^2^ the San Francisco Declaration on Research Assessment [DORA]^3^). Citation impact is only one aspect of research impact, but the fact that publication and citation numbers are easily quantifiable and have numerous registration sources has made the study and monitoring of these numbers very attractive, especially due to a lack of easy alternatives. Currently, the academic world is moving away from a reliance on bibliometrics alone, for instance, in discussions about how we recognize and reward academics. The challenge for bibliometrics is to find a use for the information it can provide in a mixed-model approach to support qualitative claims and narratives. Advancements in bibliometric analyses open up novel ways to generate valuable insights in the form of research intelligence, in which bibliometric information is used for instance to visualize networks and developments in research fields [3], or as an approach to assess societal impact [4, 5].

The Dutch UMCs are unified in the NFU (Netherlands Federation of University Medical Centres). The NFU has commissioned bibliometric reports from the CWTS for many years. In recent years the policy-makers of the UMCs and at the NFU have witnessed a decline in interest and application of the traditional citation-based metrics in policy, lobbying and research strategy. Especially for advocating the impact of the research of Dutch UMCs at the national government level, it is important to show various other aspects of research impact such as expertise, collaboration, openness and the relation between societal and scientific impact. For CWTS, there is a clear added value of finding practical implementations of bibliometric analyses and innovative ways this information is perceived and applied in strategic decision-making support.

CWTS and NFU therefore joined forces to transform the traditional bibliometric benchmarking analyses of the Dutch UMCs to answer to the shifting demands and to better showcase their own societal and scientific impact.

## Methods

In the study we have selected different sets of publications. The first set contains publications from the Dutch UMCs for the year 2018, which was used to create the first maps. This set is chosen because it still allows VOSviewer to work with the sets, as a set composed of various years would not work in the VOSviewer environment, given the quantity of publications. A second set is used for the analysis of open access publishing. As this is just an enumeration of output across open access types, in order to demonstrate the development in open access uptake, we have covered a wider range of years (2013–2018). And finally, the set to analyse the impact of Dutch UMCs compared with international benchmarks contains publications from the period 2015–2018, with additional years to analyse the citations received. At that point, 2018 was the last year of validated data by the UMCs we had access to. This allows for the necessary additional time for publications to be analysed with sufficient time to generate citation impact.

We used VOSviewer [6] to visualize research topics and collaboration networks. We project various measures such as openness of research, through bibliometrics on open access publishing, and forms of societal attention, based on social media metrics which open up the world of (clinical) guidelines, policy documentation and news media coverage, and finally, through bibliometrics on scientific collaboration, via visualization techniques and tools. The goal of this project was to highlight various forms of research impact that previously had no place in our analyses or those of others and to demonstrate the application of bibliometrics to unravel the societal relevance and impact of the scientific research of the Dutch UMCs.

The full NFU report^4^ was published in March 2021. This article will provide more conceptual background and policy considerations behind the analyses performed and will demonstrate some of the most interesting results. Specific methods used are discussed together with the results.

## Results

### Research focus and expertise

**Fig. 1:**
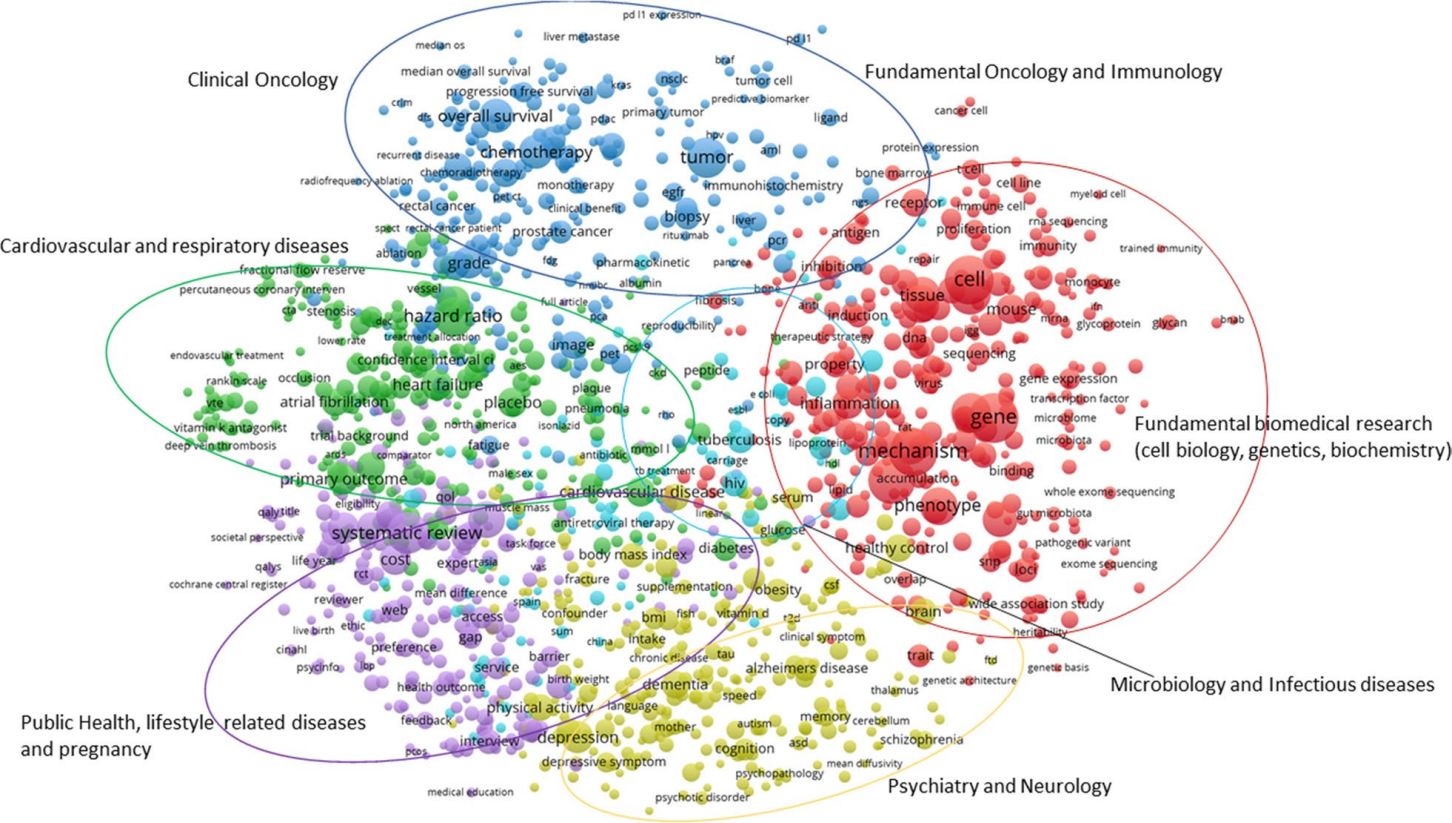
Map showing the most prominent research of Dutch UMCs in 2018

Our first goal was to create an overview of the main research streams of the Dutch UMCs to demonstrate the diversity of research. The UMCs annually provide a validated set of publications to CWTS from their own research information systems. CWTS matches these publications with their internal database linked to the Web of Science (WoS) Core Collection. To create the overview, we used the total publication set of 2018. CWTS uses a cluster algorithm to group publications on a topic level, based on co-citations [7]. This means that a publication on a certain topic will be assigned to a cluster of publications on the same topic based on which it has the strongest citation links with (both citations given and received). The benefit of these clusters is that a level playing field is created on which to perform citation-based analyses, and it also enables assessment of research focus and expert areas. To identify the major research streams in UMC research, we selected only publications in clusters with over 15 publications in 1 year, and clusters with a joint mean normalized citation score higher than 1.5 (which means 50% above world average), to combine elements of both quantity and quality that together signify a main research orientation. In the next step, VOSviewer was used to create a map (Fig. 1) of the most frequently occurring terms (in title and abstract) in the selected publication set. The sizes of the spheres in the map indicate the number of occurrences of a key term. The position and colour of the terms is indicative of their relatedness and how often they occur together in publications. VOSviewer uses standard thresholds for clustering the terms. In this case six clusters were formed, which we used to identify six main research streams in the research of the UMCs (see the annotation in Fig. 1).

From a strategic policy perspective, several observations can be made.

1. A very distinctive feature of academic medical research is the presence of a large fundamental research component. The academic medical centres invest in laboratories and infrastructure for fundamental research which forms the basis of innovations in translational and clinical research. These investments lead to clearly visible fundamental research output which distinguishes us from other hospitals. This information can be used to demonstrate the effect of, and lobby for, government funding of academic hospitals.
2. There are many interactions on closely related topics signifying the translational nature of UMC research. Fundamental research is in close contact with more clinical or public health-oriented research, and diseases are often studied from a fundamental, epidemiological, clinical and public health viewpoint. The ecosystem of a UMC, with all these dimensions under one roof, is ideal for promoting this translation. For the Netherlands this is a unique situation.
3. When the activity of each UMC is plotted onto this map, it can be observed that besides the main research streams in which they are all active, every UMC also has its own specializations. This dimension can be viewed using the interactive VOSviewer map^5^ that we built. This tool allows one to select the UMCs as a filter on the term map and look into their specific specializations. The hyper-specialization can only be achieved because the UMCs employ and enable experts on their subject areas and facilitate them with infrastructure and investments. When looking into specific clusters with very high UMC activity, we see many highly specialized topics in which the UMCs jointly excel. The maps show that the UMCs are often very complementary to each other, in the great diversity of topics they jointly study. For funders, patients and policy-makers, it is very useful to gain insight into specialization and unicity.

### Societal impact

Societal impact is a topic in the spotlight. As in the Research Excellence Framework (REF)^6^ system in the United Kingdom, societal relevance is a prominent feature in the Dutch national standard evaluation protocol (SEP^7^), which is endorsed by various main stakeholders in the Dutch academic system. As it has been quite unclear how to operationalize and showcase societal relevance, many attempts have been made in quantifying societal impact, but the fluidity of the numbers makes it very difficult to make calculations or claims. However, sources (i.e. Altmetric,^8^ Overton^9^) increasingly attempt to keep track of mentions of research in societally relevant sources such as policy documents, guidelines and news media, and the development and usability of these data for impact assessment has been investigated [8–10]. Other scholars have used bibliometric mapping software to compare the discourse of the public and researchers on Twitter and potential applications for measuring public attention [11–13].

**Fig. 2:**
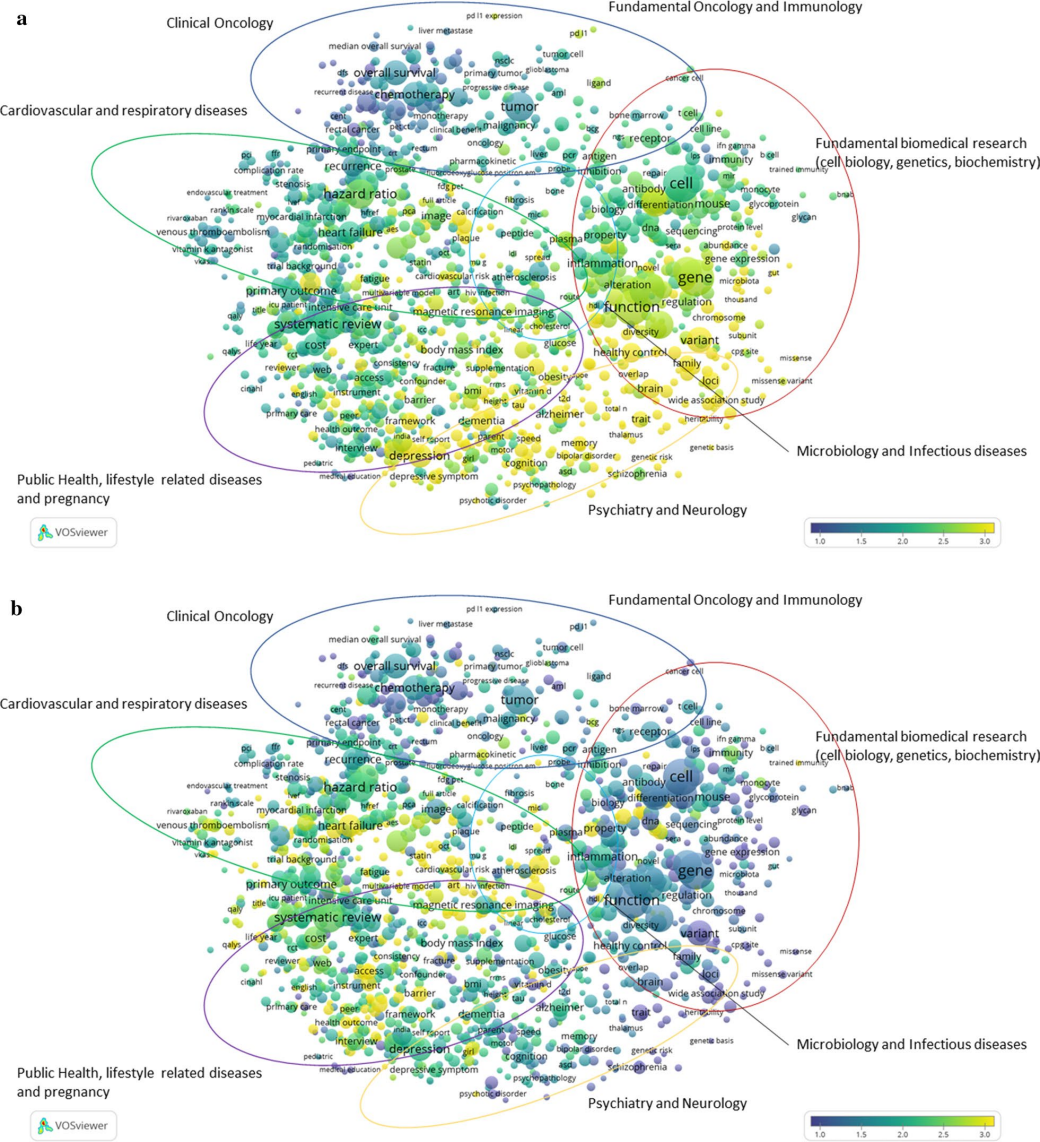

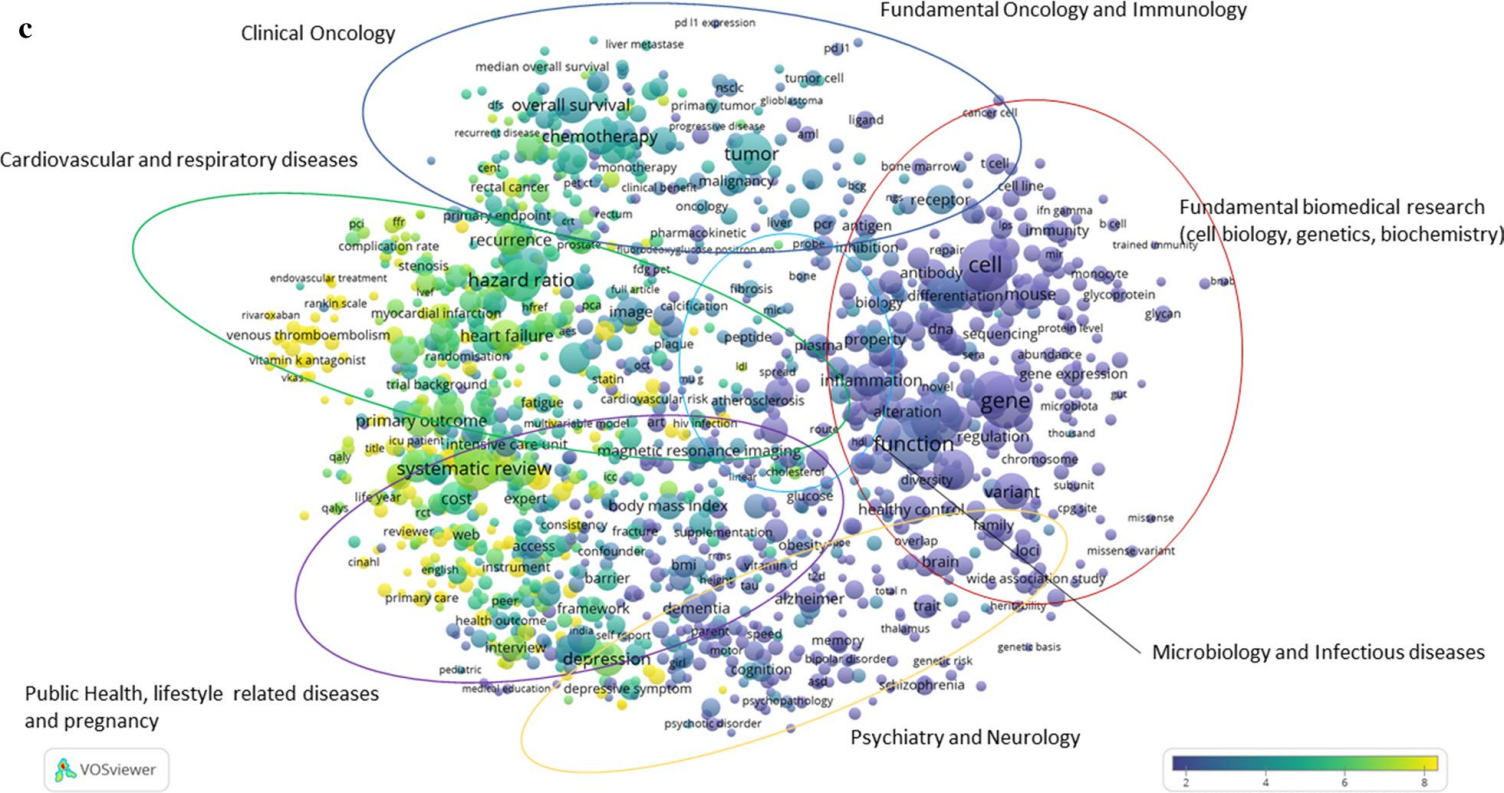
**a** Map showing the most prominent research of Dutch UMCs in 2018, as represented by news media. **b** Map showing the most prominent research of Dutch UMCs in 2018, as represented by policy documents. **c** Map showing the most prominent research of Dutch UMCs in 2018, as represented by clinical guidelines

In this approach, information from social media metrics, such as presence in policy documents, and news sites, as well as from clinical guidelines is related to the publications in the CWTS in-house WoS database. The information used to enrich the publications from the Dutch UMCs contains Digital Object Identifiers, or DOIs. Based on these DOIs, a link is created between any mention in news media, policy documents or clinical guidelines and the publications of Dutch UMCs. Thus, the explicit reference between any mention and a publication establishes such expression of relevance. By quantifying these to the level of the full set, one can display such relevance on the maps, as displayed in Fig. 2a–c. Also note that this explicit reference must be available, which means that any reference without the explicit DOI is “lost”. The dimensions are defined as follows: (1) *news media*, an indication of topics covered in newspapers, television, radio and digital media and their evidence of the direct influence of UMC research on societal knowledge and awareness; (2) *policy documents*, government documents concerning health and medicine, both national and international, and their evidence of the influence of UMC research on government strategy; (3) (*clinical*)* guidelines*, the translation of research into standard treatment procedures among medical practitioners.

The numbers on the coloured scales indicate the density of the specific social media metric in that context, in which low density is indicated by blue/purple colouring, while higher density is indicated by yellow. It varies from map to map, as the underlying information varies. This information therefore cannot be compared from one map to the other.

In Fig. 2a–c, a colour overlay is applied to show the relative percentage of news media, policy documents and clinical guidelines uptake of publications on the different topics of UMC (bio)medical research in 2018. Yellow indicates the strongest uptake by news media, policy documents or clinical guidelines, while blue indicates the lowest uptake by news media, policy documents or clinical guidelines, in Fig. 2a–c, respectively.

The information in the map does not show a score that is good or bad relative to a certain average or threshold, like a more classical bibliometric analysis could do, but it does show that Dutch UMC publications find their way to society through different channels, as well as across the different research streams. It is important to realize that the three forms of societal relevance chosen here do play different roles in the translation from the academic research realm to societal usage. In clinical guidelines, academics often still play a role, as they themselves are involved in the composition of the clinical guidelines, describing diagnosis and therapeutics on certain diseases, while the uptake in news media or policy documents is less strongly influenced by researchers and clinicians working at Dutch UMCs.

Demonstrating societal impact is increasingly important in funding applications, evaluations and the accountability of public funding. Moreover, it is a sign of a return on investment in medical research back to society by developing and improving (clinical) treatments and guidelines for practitioners, influencing policy and informing the public. We observe that, compared with the average in our publication set, clinical research often finds its way into clinical guidelines and treatments, public health-related research frequently finds its way into policy documents, and fundamental, neurological and public health research is frequently covered in news media, both national and international. These observations are very relevant because they can be used by NFU policy-makers to support claims about research impacting society, for instance, in the debate with the national government; they can also be used to strengthen funding applications by researchers in these specific subject areas, and they support narratives on societal impact in the context of periodical research evaluations. On another level, this information is also relevant for patients and citizens, because it shows where certain expertise that is relevant to them is concentrated. We applied this technique on a broad level, but it is suitable for analysing research lines in more detail as well.

### Open access to publications

**Fig. 3:**
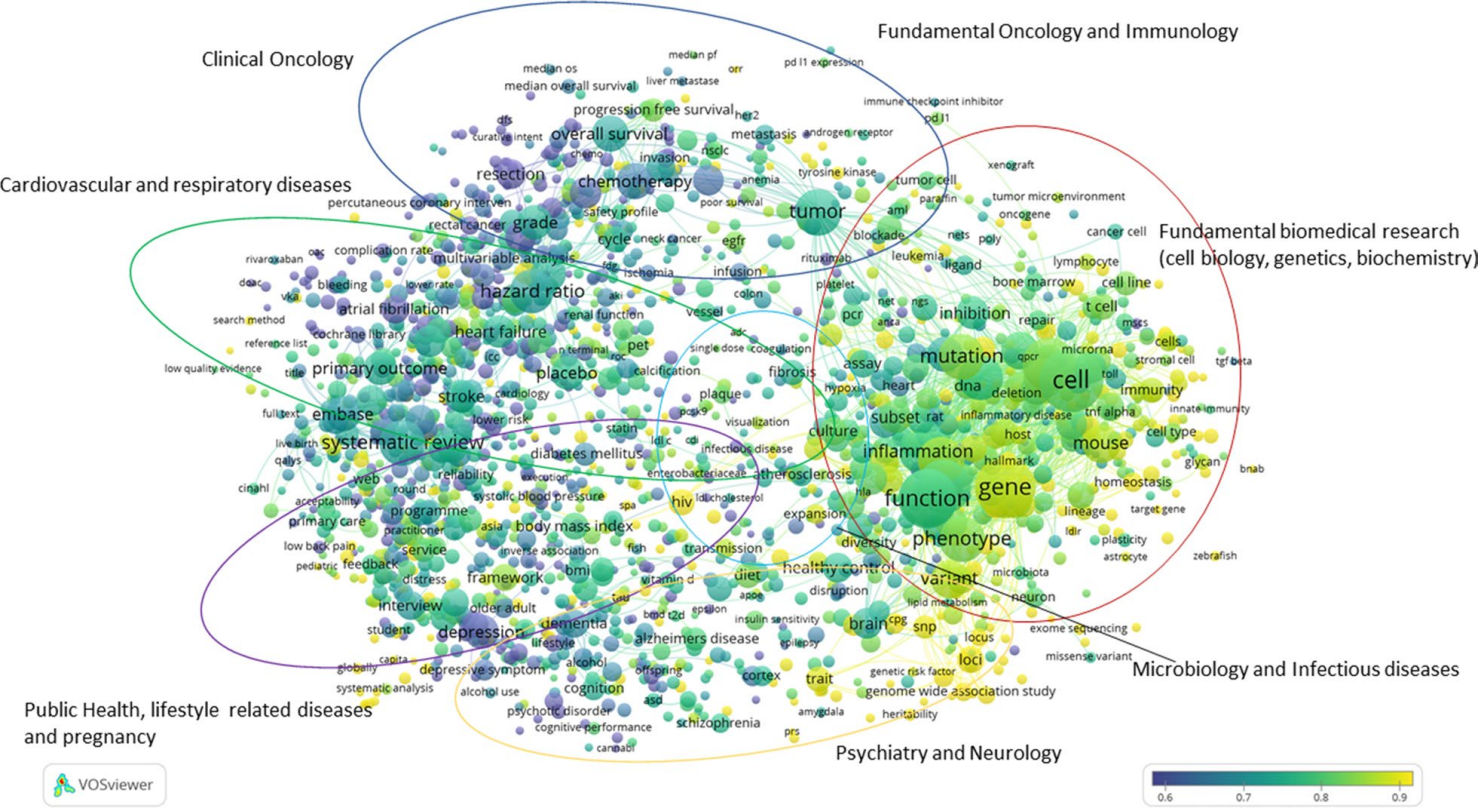
Term map of UMC publications in 2018, colours indicating relative open access of publications

In a comparable manner to visualizing the social media metrics, it is possible to plot the average open access status of publications on the previously identified topics. This map is shown in Fig. 3. It uses a colour overlay to show the relative percentage of open access of publications on the different topics of UMC (bio)medical research in 2018. Yellow is the most open, and blue the least open.

The map shows that fundamental research (right side) is more often published in open access than clinical research (top left and bottom). Also, a considerable amount of public health research with direct societal implications is published in open access (left of middle and centre). An explanation for the high percentage of open access of fundamental research is that it is common in these research communities to deposit papers in public databases such as bioRxiv^10^ or PubMed^11^ Central. Apart from that, funding agencies increasingly promote or require open access to publications that they fund (e.g. the national research council NWO, and its medical council ZonMw, as well as the European Union). In the clinical sciences, a considerable part of the research is co-funded by industry, often without the incentive to publish in open access, so this could explain the lower percentage of open access in these fields. Finally, much effort has been put into negotiating open access deals with a large share of academic publishers, which has led to more open access to publications in recent years and will continue to do so in the future.

It is our national mission to provide open access to as many scientific publications as possible, and especially to government-funded research. In total, 70% of all UMC publications in 2018 were published in open access, which is above the national average (roughly 60% in 2018). Table 1 shows the development of open access publications of the Dutch UMCs over time, and for the different types of open access.^12^ The totals of the different categories do not add up because green open access regularly overlaps with the other types of open access publishing.

**Table 1:** Development of open access publishing by all Dutch UMCs combined, 2013–2018, absolute number of publications

Open access status	2013	2014	2015	2016	2017	2018
Closed	7447 (51%)	7481 (50%)	7556 (45%)	6379 (37%)	5683 (33%)	5375 (30%)
Open access	7197 (49%)	7590 (50%)	9239 (55%)	10,740 (63%)	11,399 (67%)	12,794 (70%)
Gold	2153	2427	2937	3223	3544	4032
Green	6116	6546	7931	8780	9156	10,714
Hybrid	1039	1136	1700	2648	3062	3916
Bronze	2162	2172	2612	2972	2829	2680

### Research collaboration

Another important aspect of biomedical research is research collaboration. In the study we focused on this aspect of research over two dimensions: in the first place aiming at research collaboration with international partners, mainly academic, but not necessarily so, with the second aim of the analysis on the national context, where a variety of partners play a role. The reason for the latter analysis is mostly related to the translational dimension, whereby academic knowledge from the UMCs is transferred to the more general hospitals in the region, and also local healthcare organizations and mental health organizations, but potentially also private partners. We here show as an example the two networks of collaboration for the Erasmus Medical Centre in Rotterdam (Erasmus MC). The networks for the other UMCs can be found in the full NFU report (see footnote 6). Colour coding indicates clusters of collaborative partners, working more densely together within that cluster, as compared with partner institutes of Erasmus MC embedded in another colour-coded cluster.

**Fig. 4:**
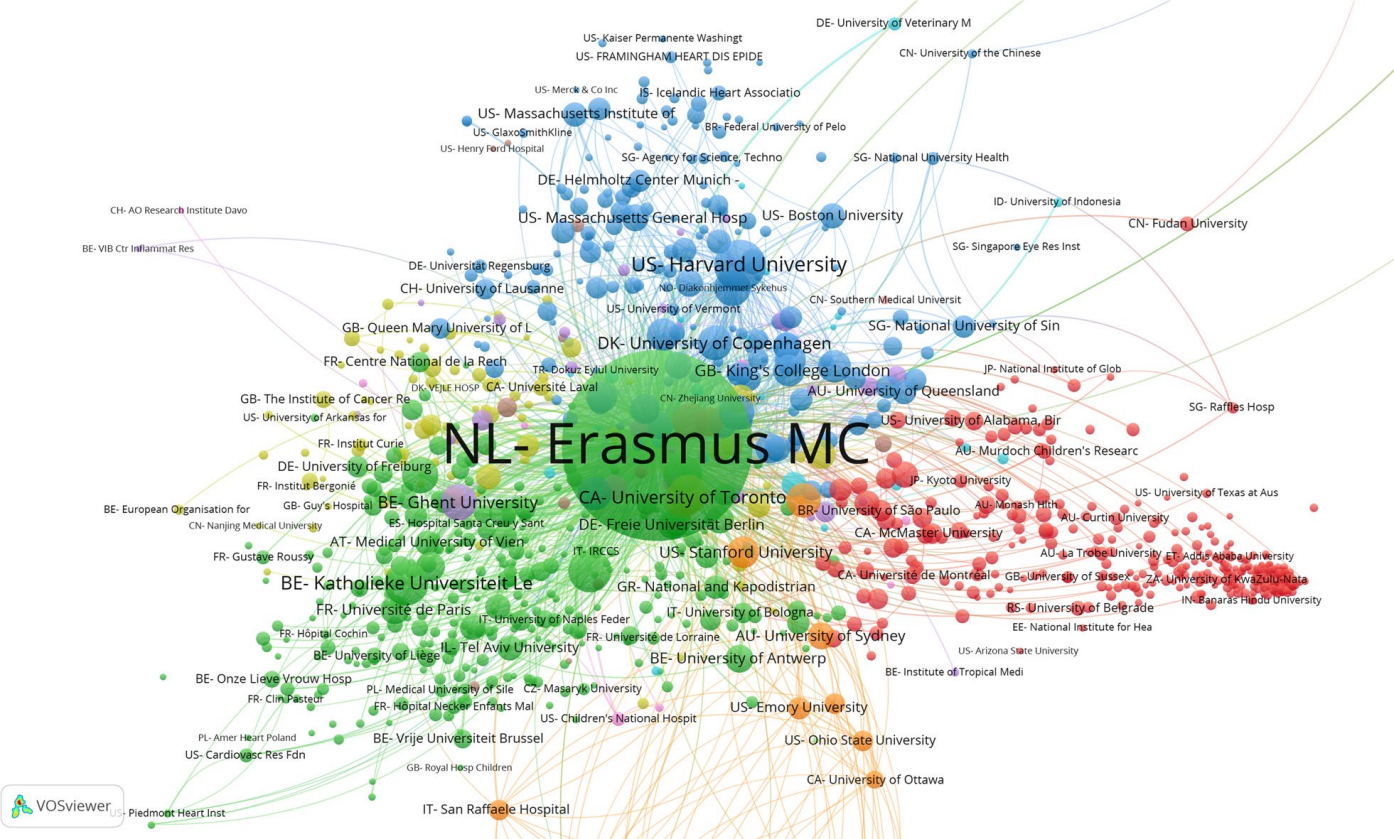
Map showing scientific cooperation with the most important international collaborators on scientific publications of Erasmus MC in 2013–2018

In Fig. 4, displaying the international scholarly cooperation, we distinguish three main clusters: the blue cluster shows that Erasmus MC has a clear connection to prominent universities in the United States, such as Harvard and Massachusetts Institute of Technology (MIT), and cooperation in the north-western region of the United States is further underpinned by the presence of Massachusetts General Hospital and Boston University. Other prominent partners here are Copenhagen University, and King’s College London in the United Kingdom. This cluster also contains several institutions focused on cardiovascular research. The green cluster shows many European universities as partners of Erasmus MC in neighbouring countries Belgium and Germany. Finally, the red cluster contains a variety of partners, from Canada, Australia, Latin America and Africa. It is important to realize that quantitative analysis of scientific cooperation can enlighten the contents of the cooperation, such as we observed in the blue cluster; it also shows that scientific cooperation is often taking place in the closer vicinity, in which neighbours play an important role [14, 15]; and finally we have to keep in mind that, increasingly, requirements related to funding, such as the European Union funding programmes Horizon 2020 and Horizon Europe, explicitly mention international scientific cooperation as an obligatory aspect of the funding procedure and, consequently, success in these procedures.

**Fig. 5:**
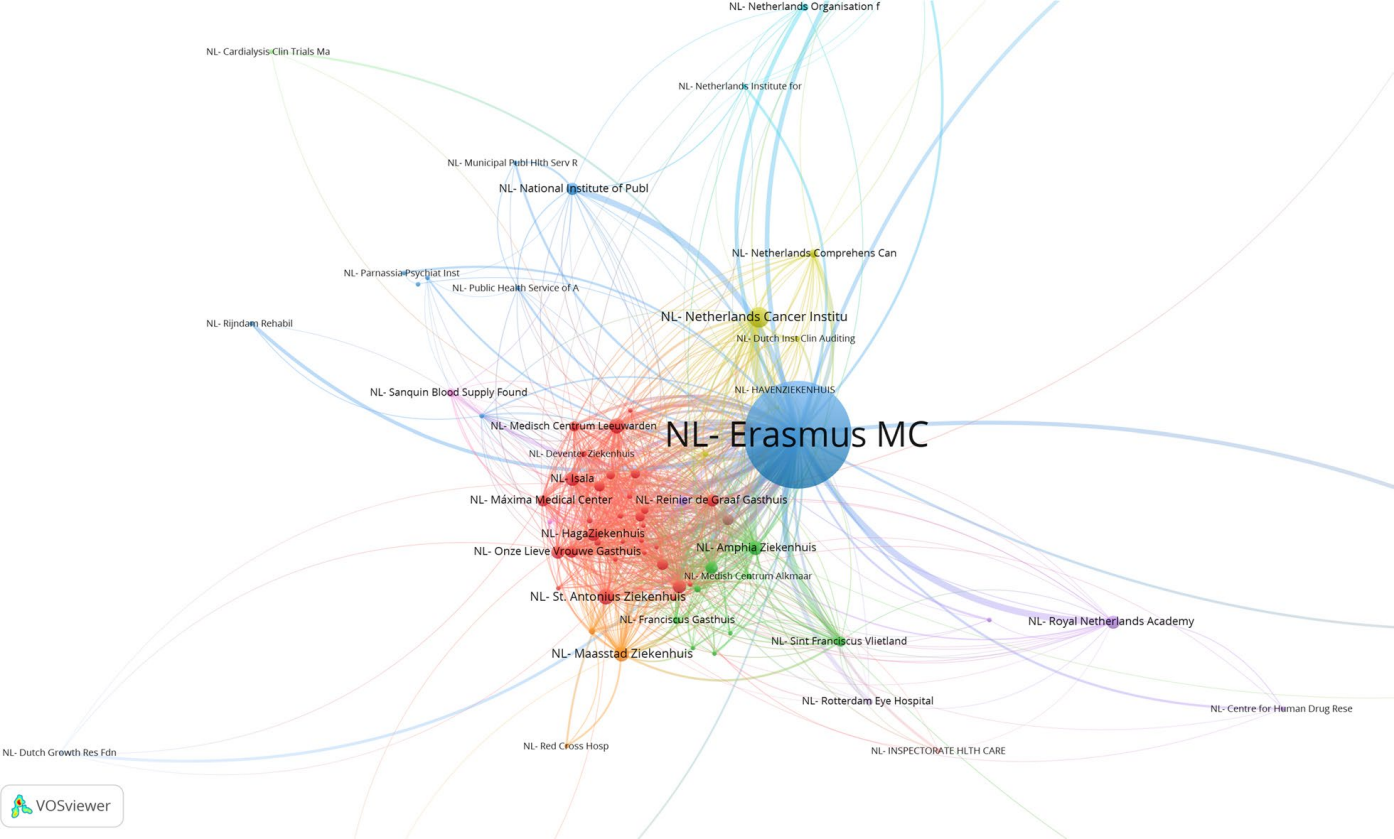
Map showing scientific cooperation with national non-university partners on scientific publications of Erasmus MC in 2013–2018

When we look at Fig. 5, which displays the scientific cooperation between Erasmus MC and Dutch nonacademic partners, we observe a few main clusters, in which groupings of Dutch peripheral hospitals cooperate with Erasmus MC. Regional and national collaboration is very important for UMCs, because this enables knowledge to flow directly into practice, and at the same time it gives researchers access to patient groups. The national government expects UMCs to operate as regional engines for medical research. In the Erasmus MC network, we can clearly see the prominent presence of regional hospitals such as the Maasstad Hospital (orange), the Amphia Hospital, the Rotterdam Eye Hospital and the Sint Franciscus Gasthuis (in the green cluster). On a national level Erasmus MC collaborates with several nonacademic hospitals (the red cluster), often in the context of large clinical trials. In the blue and yellow clusters, we see several national organizations or institutes that operate on specific medical topics (such as cancer, cardiovascular diseases, infectious diseases, rehabilitation and psychiatry). These are of course also very important partners for a UMC to collaborate with in research because of their specializations and practice-based challenges.

### Scientific impact

In the final step we put the UMC research activity and scientific impact into an international perspective, using more traditional bibliometric indicators. Publication and citation-impact data of the Dutch UMCs are compared with some of the top institutes in the biomedical field in Europe. For this comparison, publications from the period 2013–2018 were used that could be assigned to the biomedical field based on the cluster in which they were published. Affiliations were subsequently used to link those publications to the correct institutions. Many institutions in Europe do not have the same organizational structure, in which the UMC is a distinct and separate entity from the university. Therefore, we used the output of European universities active in the biomedical field as a proxy for their associated medical centres to be able to compare publication and citation-impact scores.

**Fig. 6:**
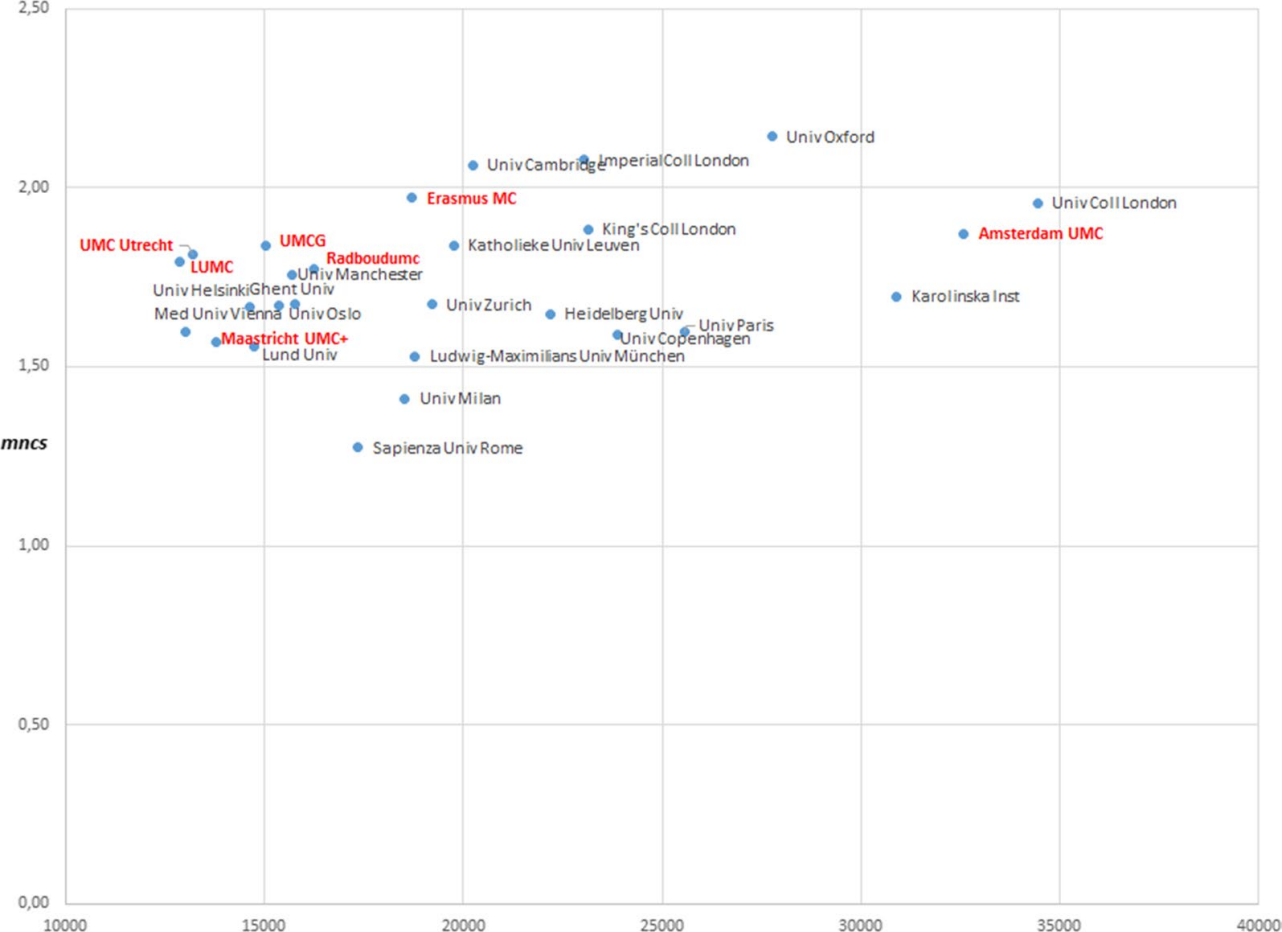
Output relative to impact (MNCs), Dutch UMCs and the top 20 European universities in biomedicine, 2013–2018

**Fig. 7:**
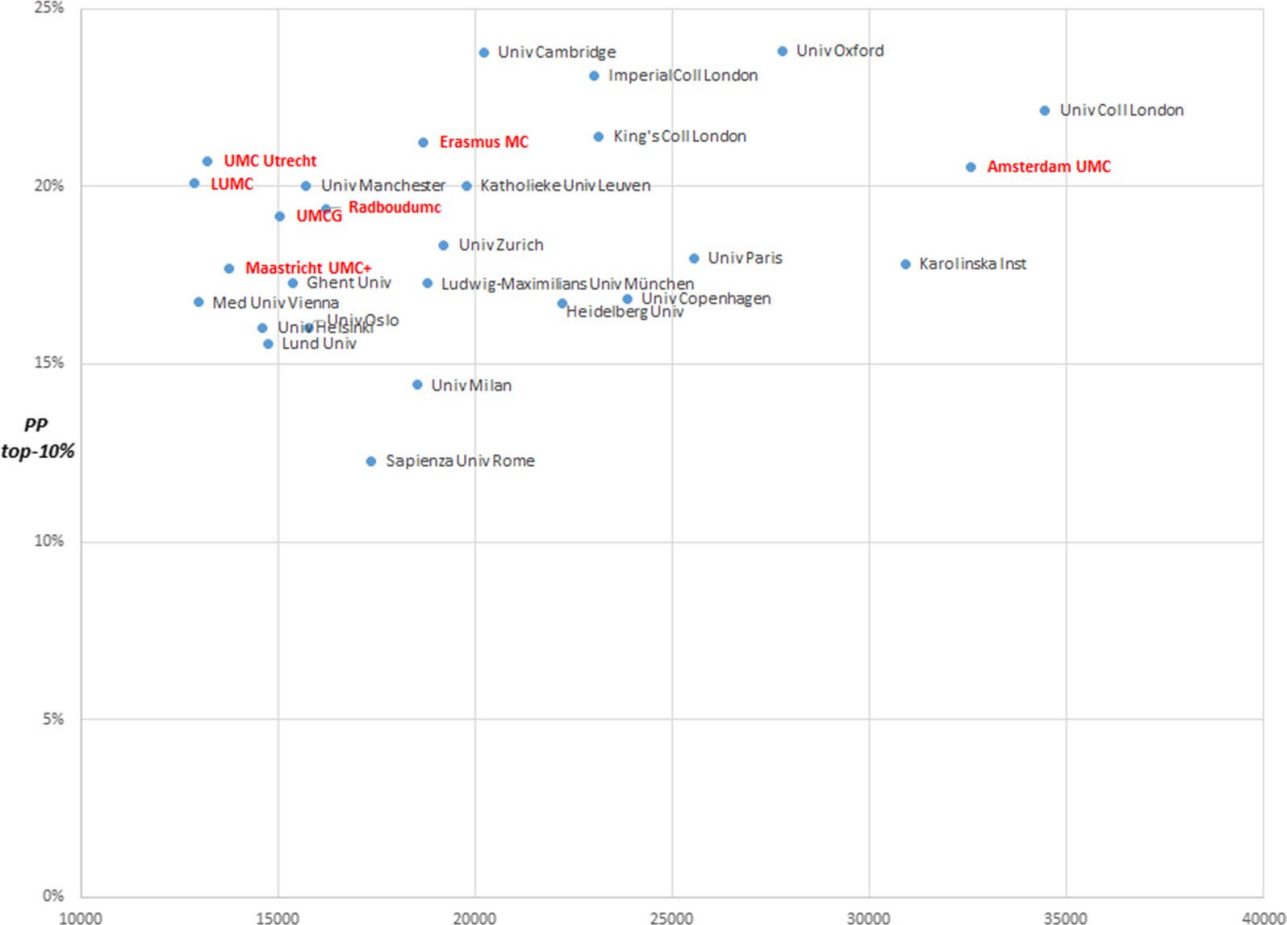
Output relative to impact (PP-top 10%), Dutch UMCs and the top 20 European universities in biomedicine, 2013–2018

Figures 6 and 7 show the mean normalized citation score (MNCS) and PP-top 10% (percentage of publications among the top 10% most highly cited publications in the same cluster) of the Dutch UMCs and the top European universities in the biomedical field on the vertical axis, and the number of publications per institute on the horizontal axis. An MNCS score of 1 reflects the world average. A higher MNCS means that publications from an institution, on average, are cited more frequently than the world average, compared with other publications in the same cluster from the same year. The merger of the Amsterdam UMCs (Academic Medical Centre [AMC] and VU University Medical Centre [VUmc]) into Amsterdam UMC makes them one of the largest biomedical research institutes based on volume of publications in the biomedical field in Europe. Irrespective of the volume of output, all Dutch UMCs are among the highest in Europe based on their scientific impact (MNCS and PP-top 10%).

## Discussion

This study reflects a development taking place on a global scale, but in particular in the Netherlands over the last 10 years—a development in which during research assessment procedures, the focus has shifted from traditional bibliometric methods, whereby output and citation analysis do play a central role, into a more contextualized approach of applying research metrics in monitoring research performance. This study has adopted that contextualized approach by blending visualizations into the methodologies, leading to a better understanding of research performance, on the contents of what has been published. Rather than ending up in benchmarking and comparing Dutch academic biomedical research over a limited set of dimensions, this approach shows the similarities, differences, strengths and collaboration possibilities as a whole. By blending in elements of societal impact, open scholarship and (inter)national scientific cooperation, the study is aimed more at the joint collective learning perspective on the basis of (the contents of) the combined research output, rather than functioning as an accountability tool, in which UMCs are compared against each other on their scientific impact only.

## Conclusions

With respect to the joint learning aspect, there are a number of main conclusions to be drawn based on the study and the visualizations and our interpretations of the maps presented in the report that provide input for national policy and strategic decisions.

The first conclusion can be summarized as “complementarity in diversity”. The research of the Dutch UMCs covers a very broad range of topics, highlighting the richness and diversity of the national biomedical landscape. While sometimes the individual UMCs may be active in the same general research fields, they specialize in topics which are very often complementary to each other. On these specific topics there is collaboration to create critical mass and impact in the international scientific world, and to optimize the translation of findings into clinical care.

The second conclusion is on the stance towards practicing open science. It is the Dutch national mission to strive towards a high level of openness, and in particular to open access to Dutch UMCs’ scientific publications. Our analyses show that the Dutch UMCs have taken this message to heart, and even in 2018, some 70% of the research papers can be accessed openly and freely by anyone. Because of this, researchers around the world, healthcare practitioners and the general public should benefit directly from the research conducted by Dutch UMCs. This is especially important since the largest part of the research conducted was government-funded. Society should be able to reapply this knowledge in their own research or practices in order to accelerate developments in medical research and healthcare.

The analyses further show that the Dutch UMCs operate on a world-class level, collaborating in research with top institutions around the globe. UMC research scores at the international top, in terms of both volume and citation impact. With limited governmental expenditure^13^ compared with other countries based on the gross domestic product (GDP), the Dutch UMCs publish many papers that are valued by peers.

In addition to their prominent and leading role in the international research landscape, the Dutch UMCs are the scientific driver of biomedical research in the Netherlands (nationally and regionally). Each UMC has a distinct national collaboration network, naturally including universities, but also scientific organizations, regional hospitals and industry partners. Each UMC plays a leading and coordinating role in research with these regional partners. This is a symbiotic relationship in which all partners have their unique roles, and in which knowledge and expertise are exchanged.

With respect to recommendations to others, the study shows how one can shift away from a classical bibliometric analysis into a mode in which a variety of different approaches and techniques offer many new insights for research management and science policy-making. Adding open access data and social media mentions to publication outputs gives new insights into the developments of Dutch academic biomedical research and the way it is being picked up in other forms of communication. By focusing on cooperation and complementarity instead of competition and differences, one can see how such characteristics can be useful in understanding how a national academic biomedical research system functions and operates.

In the Netherlands, academic institutions perform evaluations of their research using a continuously developing SEP. This protocol currently offers considerable freedom to make choices on how a unit wants to be evaluated. The discussion on research evaluation is open, following a broader perspective on how to handle this evaluation and the monitoring of research performance. This study used this freedom to sketch the landscape the UMCs find themselves in, from the perspective of collaboration and analyses of open science. It shows that the Dutch medical sciences serve not only their peer community but also the general population through different channels. Our assignment was to analyse how the UMCs collaborate instead of comparing them numerically with each other, and provide intelligence on the entire collaboration networks, the content and complementarity of the work, and different dimensions besides the impact in academic peer groups. Our approach uses mixed models to move away from a strictly numerical valuation towards a broader perspective of the landscape we operate in. To conclude, in this work we highlight the different aspects of the research impact of the Dutch UMCs using bibliometric techniques in a novel way to generate research intelligence. At the base of the analysis were a number of strategic questions, which were designed by policy advisors and translated into an analytic approach by the research intelligence experts in close contact with the bibliometric expert. Because of this intense collaboration and efficient exchange of ideas, we were able to interpret the bibliometric analyses and present them in such a way that they could be directly used by the policy advisors in their work on a national level. With this information, they are able to show the strength of the combined force of Dutch biomedical research, and its significant impact, which is for instance crucial in debates on national investments in biomedical research.

## Data Availability

Not applicable.

## Abbreviations

UMC: University medical centre
CWTS: Centre for Science and Technology Studies, Leiden
NFU: Netherlands Federation of University Medical Centres
WoS: Web of Science
REF: Research Excellence Framework (United Kingdom)
SEP: Standard evaluation protocol
MNCS: Mean normalized citation score
GDP: Gross domestic product

## Footnotes

1 http://www.leidenmanifesto.org/.

2 https://responsiblemetrics.org/the-metric-tide/.

3 https://sfdora.org/read/

4 https://www.nfu.nl/sites/default/files/2021-03/Research_Impact_of_the_Dutch_University_Medical_Centres.pdf.

5 https://www.nfu.nl/themas/onderzoek-en-innovatie/impact-van-onderzoek/onderzoeksonderwerpen.

6 https://www.ref.ac.uk/.

7 https://www.universiteitenvannederland.nl/en_GB/sep-eng.html.

8 https://www.altmetric.com/.

9 https://www.overton.io/.

10 https://www.biorxiv.org/.

11 https://www.ncbi.nlm.nih.gov/pmc/about/intro/.

12 https://www.cwts.nl/blog?article=n-r2w2a4.

13 https://www.rathenau.nl/en/science-figures/investments/international-perspective-rd-investments/rd-investments-international.
